# Toll-Like Receptor 4 Mediates Endothelial Cell Activation Through NF-κB but Is Not Associated with Endothelial Dysfunction in Patients with Rheumatoid Arthritis

**DOI:** 10.1371/journal.pone.0099053

**Published:** 2014-06-11

**Authors:** Rossella Menghini, Umberto Campia, Manfredi Tesauro, Arianna Marino, Valentina Rovella, Giuseppe Rodia, Francesca Schinzari, Barbara Tolusso, Nicola di Daniele, Massimo Federici, Angelo Zoli, Gianfranco Ferraccioli, Carmine Cardillo

**Affiliations:** 1 Department of System Medicine, University of Tor Vergata, Rome, Italy; 2 Division of Cardiology, MedStar Heart Institute, Washington, DC, United States of America; 3 Department of Internal Medicine, Catholic University Medical School, Rome, Italy; 4 Department of Rheumatology, Catholic University Medical School, Rome, Italy; 5 Center for Atherosclerosis, Policlinico Tor Vergata, Rome, Italy; INRCA, Italy

## Abstract

**Objective:**

To investigate the effects of TLR4 antagonism on human endothelial cells activation and cytokine expression, and whether the Asp299Gly TLR4 polymorphism is associated with better endothelial function in patients with rheumatoid arthritis (RA).

**Methods:**

Human aortic endothelial cells (HAECs) were treated with lipopolysaccharide (LPS), OxPAPC, and free fatty acids (FFA) at baseline and after incubation with the TLR4 antagonist eritoran (E5564). Cytokine expression was assessed by quantitative real-time PCR. In vivo endothelial function was assessed as brachial artery flow-mediated dilation (FMD) in RA patients with the wild type gene (aa) and with the Asp299Gly TLR4 polymorphic variant (ag).

**Results:**

In HAEC, TLR4 antagonism with eritoran inhibited LPS-induced mRNA expression of IL-6, IL-8, TNFα, CCL-2, VCAM and ICAM (P<0.05 for all) and inhibited Ox-PAPC-induced mRNA expression of IL-8 (P<0.05) and IL-6, albeit not to a statistically significant level (p = 0.07). In contrast, eritoran did not affect FFA-induced mRNA expression of IL-6 (P>0.05). In 30 patients with RA (15 with the ag allele) undergoing measurement of FMD, no differences in FMD and plasma levels of IL-6, IL-8, VCAM, and ICAM were found between the aa and the ag phenotype (P>0.05 for all).

**Conclusions:**

TLR4 signaling in endothelial cells may be triggered by LPS and oxidized phospholipids, leading to endothelial activation and inflammation, which are inhibited by eritoran. Our in vivo investigation, however, does not support an association between the Asp299Gly TLR4 polymorphism and improved endothelium-dependent vasodilator function in patients with RA. Further study is needed to better understand the potential role of TLR4 on endothelial dysfunction in this and other patient populations.

## Introduction

Chronic inflammation represents a pivotal mechanism in the pathogenesis of atherosclerosis [1]. Interestingly, recent evidence suggests that innate immunity may also contribute to the development of vascular damage by interacting with inflammatory pathways [2]. In particular, toll-like receptors (TLRs) are increasingly being recognized as a link between the innate immune system, inflammation, and atherogenesis. This family of innate immune receptors is expressed by endothelial cells, in which they trigger various signaling pathways and lead to cell activation, increased expression of inflammatory cytokines and adhesion molecules, and endothelial dysfunction [3], [4]. While initially identified as sensors of microbial invasion, TLRs are now known to be activated also by endogenous ligands produced in inflamed tissues, potentially leading to further inflammation and perpetuating an inflammatory milieu [3]. Among them, TLR4, a receptor for lipopolysaccharide (LPS) from Gram negative bacterial cell walls, also exhibits affinity for fatty acids [5], extracellular matrix components, fibrinogen, and various heat shock proteins [6]. Of note, TRL4 signaling leads to activation of NF-κB [4], a pathway associated with andothelial injury [7], and TRL4 expression is increased in human atherosclerotic plaques [8]. Additionally, lack of TLR4 reduces atherosclerosis and alters plaque phenotype in apoE-deficient mice fed a high-cholesterol diet [9]. In agreement with these data, clinical evidence indicates that the Asp299Gly TLR4 polymorphism, a functional variant in the TLR4 gene (896A→G) that attenuates receptor signaling and diminishes the inflammatory response to LPS [10], is associated with decreased atherosclerotic risk [11]. However, whether antagonism of TLR4 prevents TLR4-induced expression of inflammatory cytokines and adhesion molecules in human macrovascular endothelial cells has not been investigated in detail.

Rheumatoid arthritis (RA) is one of the most prevalent systemic autoimmune diseases [12] and is associated with endothelial dysfunction [13] and increased cardiovascular risk [14]. This risk is attributed to the presence of both traditional and non-traditional risk factors, including inflammation and immunologic abnormalities [15]. A growing body of knowledge indicates that TLR4 may play a relevant role of in the pathogenesis of autoimmune damage in RA [3]. In line with this evidence, the Asp299Gly TLR4 polymorphism is associated with decreased RA disease susceptibility and lower baseline disease activity [16]. However, whether the presence of the Asp299Gly TLR4 polymorphism is associated with better endothelial function compared with the wild type genotype in patients with RA has not been studied.

The current investigations were therefore designed to test the following hypotheses: 1) antagonism of TLR4 with eritoran (E5564) inhibits the expression of inflammatory cytokines and adhesion molecules in human endothelial cells; and 2) the presence of the Asp299Gly TLR4 polymorphism is associated with better endothelium-dependent vasodilation compared with the wild type genotype in patients with RA.

## Materials and Methods

### In-Vitro Experiments: Cell Culture and Treatment

Human aortic endothelial cells (HAECs) were purchased from Lonza (Basel, Switzerland) and cultured according to the manufacturer’s instructions. All experiments were performed using HAECs between the 2^th^ and the 5^th^ passage. HAECs were treated with: LPS (Sigma Aldrich, St. Louis, MO) at a concentration of 100 ng/mL for 6 hours; ox-PAPC (oxidation products of 1-palmitoyl-2-arachidonoyl-sn-glycerol-3-phosphatidylcholine, Hycult Biotech, Uden, The Netherlands), an antigenic epitope of oxidized LDL, at a concentration of 100 µg/mL for 6 hours; or long chain free fatty acids (FFA, oleic acid 500 µM+palmitic acid 500 µM) for 24 hours. Cells were incubated with 10 nM eritoran for 30 minutes prior to treatments where indicated. Eritoran (Eisai Inc., Woodcliff Lake, NJ) is a synthetic analog of lipid A and a potent and specific antagonist of LPS action, which inhibits lipid A binding to MD2 and terminates MD2/TLR4-mediated signaling [17]. At the end of treatments, HAECs where collected and used for molecular analysis.

### In-Vitro Experiments: Gene Expression Analysis

Total RNA was isolated from HAECs using Trizol reagents (Invitrogen Corp, Eugene, OR). A total of 2 µg of RNA was reverse-transcribed into complementary DNA (cDNA) using the High Capacity cDNA Archive kit (Applied Biosystems, Foster City, CA). Fifty nanograms of cDNA was amplified by real-time polymerase chain reaction (RT-PCR) using an ABI PRISM 7500 System and TaqMan reagents (Applied Biosystems) and normalized to 18S ribosomal RNA as an endogenous control. Each reaction was performed in triplicate, and the relative gene copy number was calculated as 2^−ΔΔCt^ as previously described [18].

### In-Vitro Experiments: Western Blots

Total protein was isolated from HAECs and western blots were performed as previously described [19]. The following antibodies were used: anti-P65, anti-phosphoSer^536^ P65, anti-IκBα, and anti-phosphoSer^32/36^ IκBα, (Cell Signaling Technology Inc., Danvers, MA).

### Clinical Study: Study Population

Nonsmoker patients with a diagnosis of RA according to the ACR revised criteria [20] and age- and sex-matched healthy controls were enrolled in the study. None of the patients had history or presence of hypertension, diabetes, hypercholesterolemia, cardiovascular disease, vasculitis, or any other systemic condition. All RA patients were on chronic treatment with either disease modifying antirheumatic drugs (DMARDs), monoclonal antibodies, or their combination. When used, aspirin, coxibs or other nonsteroidal anti-inflammatory drugs were withdrawn at least one week before the study; patients were allowed to use acetaminophen (paracetamol) or tramadol as needed. The Disease Activity Score 44 (DAS 44) was calculated for each patient. This score assesses disease activity by including tender and swollen joint count and the erythrocyte sedimentation rate. The level of disease activity can be interpreted as low (DAS≤2.4), moderate (2.4<DAS≤3.7), or high (DAS>3.7) [21]. A DAS<1.6 corresponds with being in remission according to the American Rheumatism Association (ARA) criteria [22]. The study protocol was conducted according to the principles expressed in the Declaration of Helsinki, was approved by the institutional Ethics Committee of Tor Vergata University, and all participants gave written informed consent.

### Clinical Study: Laboratory Methods

EDTA-collected blood samples were drawn on the study day after an overnight fast, immediately centrifuged for 15 min at 1000×g and stored at −80°C until analysis.

#### Detection of autoantibodies

Rheumatoid factor (RF)-IgM and RF-IgA (Orgentec Diagnostika GmbH, Mainz, Germany) and anti-CCP antibodies (Axis Shield Diagnostics, Dundee, UK) were measured using commercially available ELISA kits Equal volumes for each sample were loaded according to each specific kit’s instructions. The suggested cut-off levels were 20 U/mL for RF-IgM and RF-IgA and 5 U/mL for anti-CCP antibodies. *Soluble biomarkers:* plasma levels of IL-6, IL-8, ICAM, VCAM and MCP-1 were measured using commercially available ELISA kits (R&D Systems, Minneapolis, MN, USA). Equal volumes for each sample were loaded according to each specific kit’s instructions. Quantitative levels of cytokines were determined by comparison with standard curves and reported as picograms or nanograms per mL (pg/mL or ng/mL). The sensitivity of the test was of 0.7 pg/mL for IL-6, 1.5 pg/mL for IL-8, 0.1 ng/mL for ICAM, 0.6 ng/mL for VCAM and 5.0 pg/mL for MCP-1. Genomic DNA for TLR4 genotyping was prepared from frozen whole blood with the use of a blood DNA isolation kit (Genomic Prep, Amersham Pharmacia Biotech, Piscataway, NJ). Subsequent allele-specific PCR amplification for the TLR4 allele Asp299Gly was performed according to a previously described protocol [23].

### Clinical Study: Endothelial Function Testing

Assessment of endothelial function was conducted in the fasting state using a standardized ultrasound procedure [24]. Brachial artery reactivity, a test of endothelium-dependent vasodilation, was assessed as previously reported [25]. Briefly, participants lay supine on a bed and were allowed to rest for at least 10 minutes. During the rest period, participants were connected to a continuous ECG monitor and a pressure cuff was applied around the upper forearm. The left brachial artery was then visualized on the anterior aspect of the arm, 2–15 cm proximal to the antecubital fossa, using a Logiq E ultrasound machine (GE Healthcare Italia, Milan, Italy) with a high-resolution probe (12-MHz linear array transducer). After baseline images and flow measurements were obtained, the pressure cuff applied on the forearm was inflated at 250 mmHg for 5 minutes. Blood flow was measured during the first 15 seconds after cuff deflation, and arterial image acquisition for diameter measurements was performed between 60 and 90 seconds after cuff deflation. Arterial diameter was measured from the anterior to the posterior interface between the lumen and the endothelium at end diastole, incident with the R wave on the ECG. Images were analyzed by an investigator, different from the sonographer, blinded to image sequence and clinical data of study participants.

### Statistical Analysis

For the clinical study, sample size calculation was based on differences between the values of FMD in the three groups. Using a 2-sided paired t test, a sample of 12 subjects in each group was calculated to be necessary to detect a 2% difference in FMD with 80% power and α<0.05. With anticipation of up to 3 (i.e. 20%) technically inadequate vascular studies, 15 participants per group were enrolled to yield 12 evaluable participants in each group. All group data are reported as mean ± SD. Group differences were analyzed by one way ANOVA, Fisher exact test, and unpaired Student t test, as appropriate. All calculated p values are two-tailed, and a value of p<0.05 was considered to indicate statistical significance. Statistical analyses were performed using commercially available software.

## Results

### In-Vitro Experiments: Eritoran Inhibits LPS-Induced mRNA Expression of Inflammatory Cytokines in HAECs

**Figure 1:**
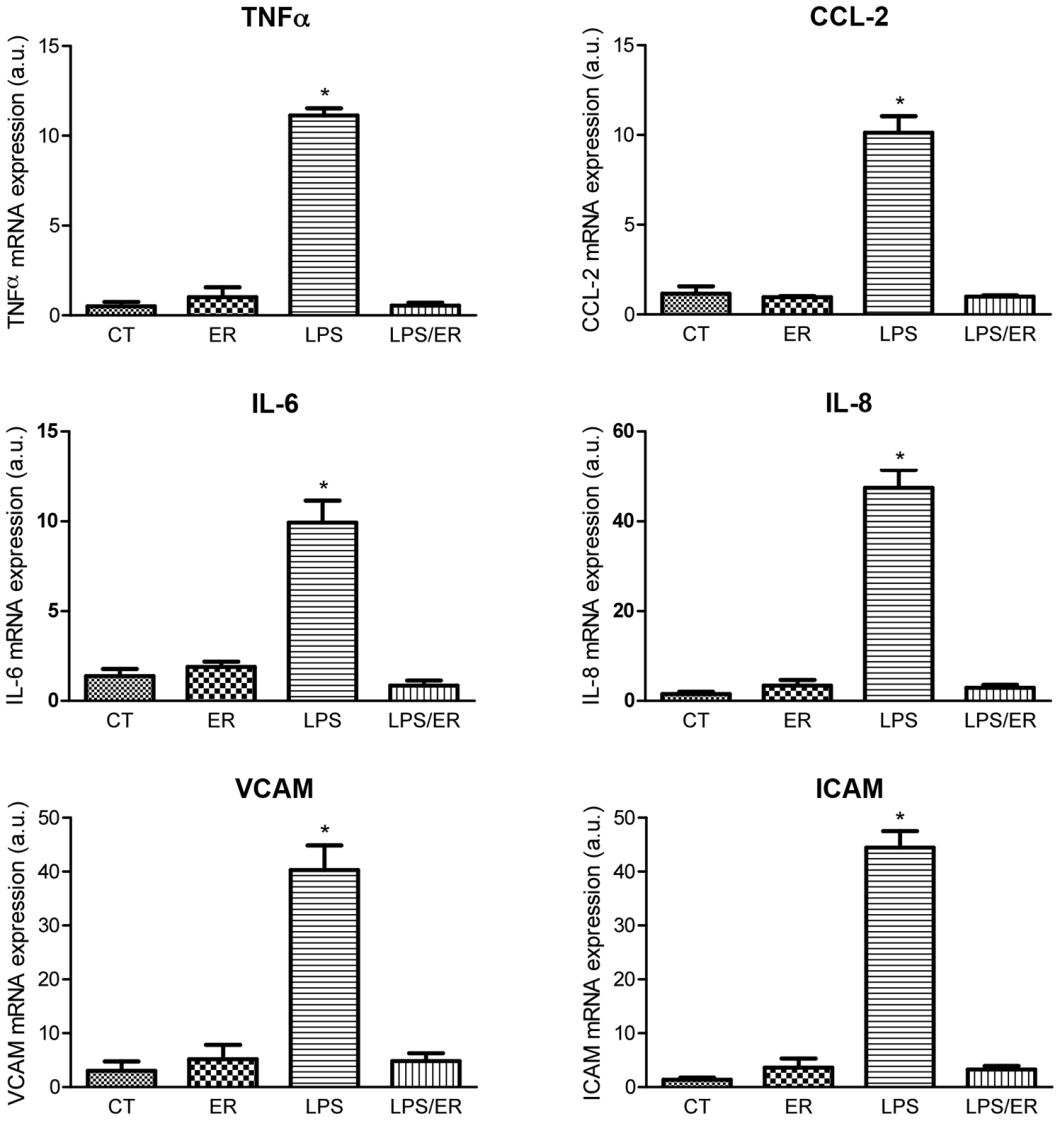
Effects of eritoran on LPS-induced mRNA expression (arbitrary units) of TNFα (top left panel), CCL-2 (top right panel), IL-6 (middle left panel), IL-8 (middle right panel), VCAM (bottom left panel), and ICAM (bottom right panel). Values reported as mean±SD (n = 5 per group). CT: control; ER: eritoran; LPS: lipopolysaccharide; ER/LPS: eritoran/lipopolysaccharide. *p<0.05 vs CT, ER, and LPS/ER.

To determine the effects of TLR4 receptor antagonism on LPS-induced expression of inflammatory cytokines in HAECs, we assessed mRNA levels of IL-6, IL-8, TNFα, and CCL-2 mRNA after treatment with LPS, alone and following pretreatment with eritoran for 30 minutes. Incubation with eritoran did not lead to significant changes in mRNA levels of these cytokines compared to control. Treatment with LPS alone for 6 hours induced a significant increase in cytokine expression. In contrast, when HAECs were treated with LPS following pretreatment with eritoran, cytokine mRNA levels were similar to control (figure 1, top and middle panels).

### In-Vitro Experiments: Eritoran Inhibits LPS-Induced mRNA Expression of Adhesion Molecules in HAECs

To assess whether TLR4 receptor antagonism impacts LPS-induced expression of adhesion molecules in HAECs, we measured mRNA levels of VCAM and ICAM after treatment with LPS, alone and following pretreatment with eritoran for 30 minutes. compared with control, eritoran alone did not lead to significant changes in VCAM and ICAM mRNA levels. Treatment with LPS alone for 6 hours caused a significant increase in mRNA levels of the adhesion molecules. In contrast, when HAEC were treated with LPS following pretreatment with eritoran, mRNA levels of VCAM and ICAM were similar to control (figure 1, bottom panels).

### In-Vitro Experiments: Eritoran Inhibits ox-PAPC-Induced mRNA Expression of IL-8 in HAECs

**Figure 2:**
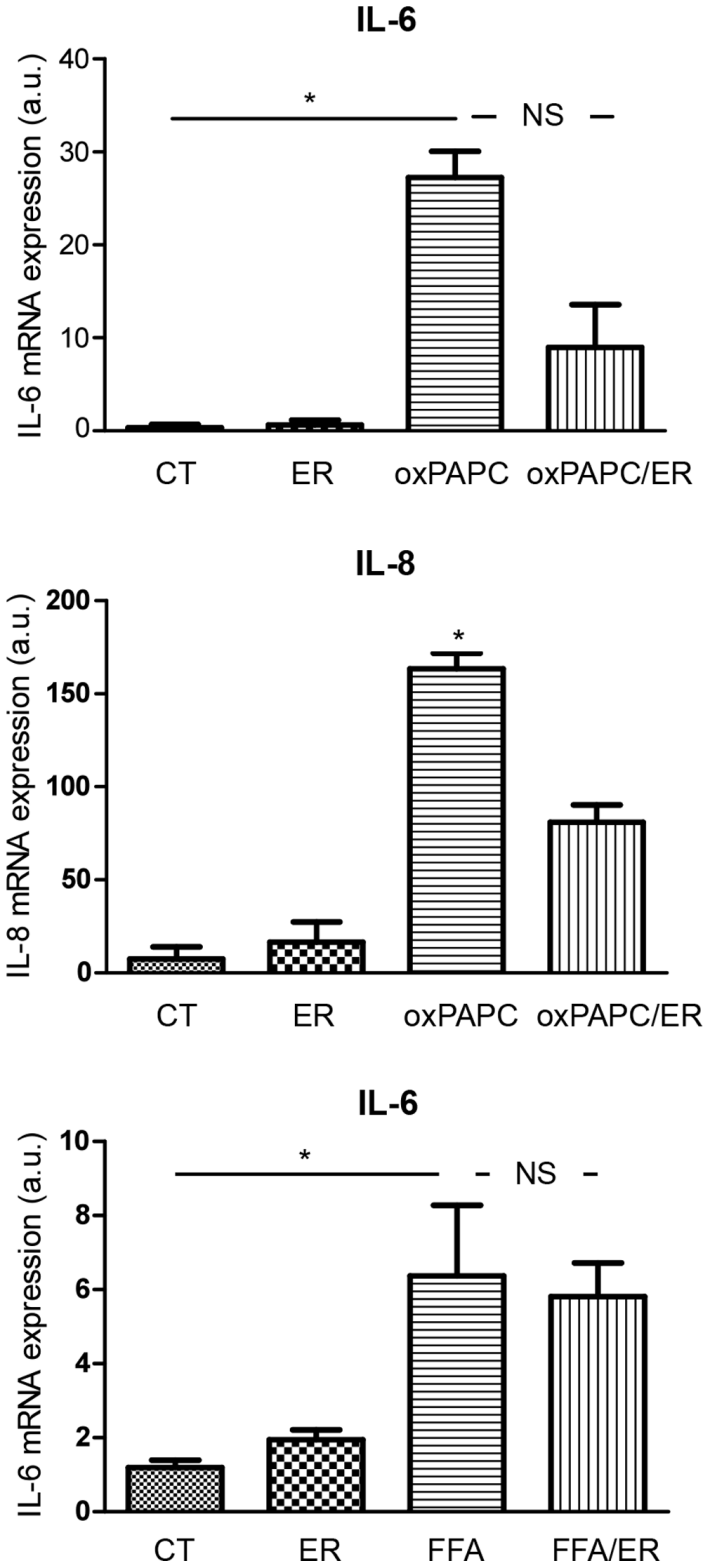
Effects of eritoran on OxPAPC-induced mRNA expression (arbitrary units) of IL-6 (top panel) and IL-8 (middle panel) and on FFA-induced mRNA expression (arbitrary units) of IL-6 (bottom panel). Values reported as mean±SD (n = 5 per group). CT: control; ER: eritoran; OxPAPC: oxidation products of 1-palmitoyl-2-arachidonoyl-sn-glycerol-3-phosphatidylcholine; FFA: free fatty acids; OxPAPC/ER: oxidation products of 1-palmitoyl-2-arachidonoyl-sn-glycerol-3-phosphatidylcholine/eritoran. Top panel: *p<0.05 vs CT and ER; NS: p = 0.07 vs OxPAPC/ER. Middle panel: *p<0.05 vs CT, ER, and OxPAPC/ER. Bottom panel: *p<0.05 vs CT and ER; NS: p>0.05 vs FFA/ER.

To explore the effects of TLR4 receptor antagonism on ox-PAPC-induced expression of inflammatory cytokines in HAECs, we assessed the effects of ox-PAPC treatment on mRNA levels of IL-6 and IL-8, alone and following pretreatment with eritoran for 30 minutes. Eritoran did not affect cytokine mRNA levels compared with control. ox-PAPC alone for 6 hours significantly increased cytokine mRNA levels. In contrast, when ox-PAPC treatment followed pretreatment with eritoran, mRNA levels of IL-8 were significantly reduced when compared with ox-PAPC alone (figure 2, top panel). Similarly, mRNA levels of IL-6 were reduced; however, they failed to reach statistical significance (p = 0.07) (figure 2, middle panel).

### In-Vitro Experiments: Eritoran Does Not Affect FFA-Induced mRNA Expression of IL-6 in HAECs

To confirm that eritoran’s effects on inflammatory cytokines are mediated by TLR4, we assessed the effects of FFA treatment on mRNA levels of IL-6, alone and after incubation with eritoran for 30 minutes. As in the previous experiments, eritoran did not affect IL-6 mRNA levels compared with control. FFA alone for 6 hours led to significantly increased IL-6 mRNA levels. Consistent with our hypothesis that eritoran’s effects on inflammatory cytokines are mediated by TLR4, pre-treatment with eritoran did not affect FFA-induced inflammatory cytokine expression (figure 2, bottom panel).

### In-Vitro Experiments: Eritoran Reduces LPS-Induced Phosphorylation of NF-κB p65 Subunit and IκB-α in HAECs

**Figure 3:**
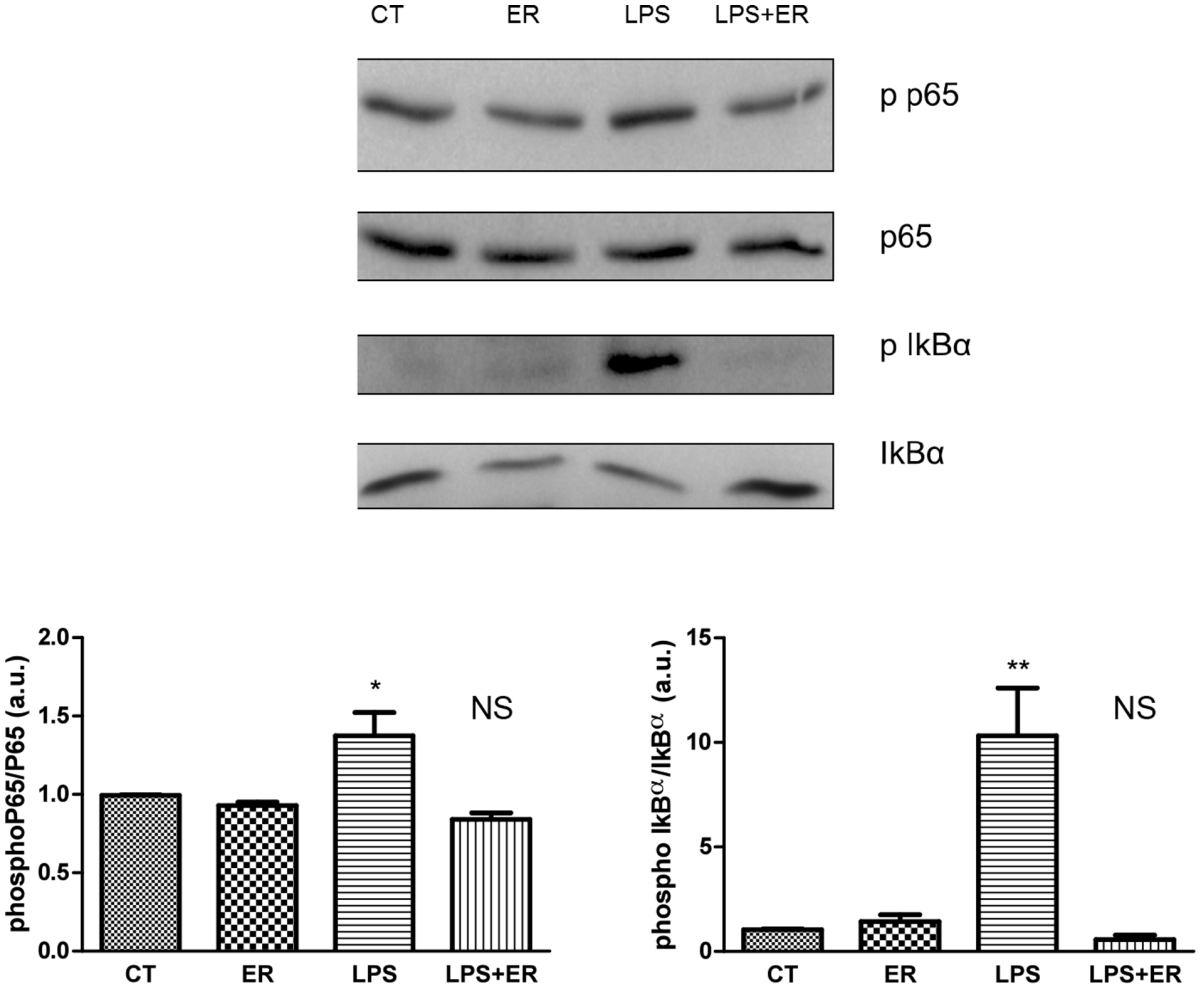
Representative Western blots (top panel) and bar graphs showing the effect of eritoran on LPS-induced phosphorylation of NF-κB p65 subunit (bottom left panel) and IκB-α (bottom right panel) (n = 5 per group). Bottom left panel: *p<0.05 vs CT; NS: p>0.05 vs LPS/ER. Bottom right panel: **p<0.005 vs CT; NS: p>0.05 vs LPS/ER.

To confirm that the effects of TLR4 antagonism on the expression of inflammatory cytokines are mediated by a modulation of the NF-κB pathways, we assessed phosphorylation of NF-κB p65 subunit and of IκB-α by western blotting (figure 3, top panel). Compared with control, treatment with eritoran for 30 minutes did not induce phosphorylation of NF-κB p65 subunit and of IκB-α. In contrast, treatment of HAECs with LPS for 6 hours caused an increase in the phosphorylation of NF-κB p65 subunit and IκB-α. Pretreatment with eritoran prevented the LPS-induced phosphorylation of NF-κB p65 subunit (figure 3, bottom left panel) and IκB-α (figure 3, bottom right panel).

### Clinical Study: Study Population

Thirty patients and 15 age- and sex-matched healthy controls took part in the study. Their baseline clinical characteristics are reported in Table 1. The mean DAS 44 score of all the patients was 2.2 (range: 0.5–3.9). The majority of the patients were treated with a disease-modifying anti-rheumatic drug (27/30) and/or a biologic agent (19/30). No significant differences were observed in these parameters, disease activity, and drug treatment between the two patient groups (all P>0.05).

**Table 1:** Baseline Characteristics of Study Participants.

Parameter		TLR4 Alleles		P
	Control	AA (Wild type)	AG (Asp299Gly)	
***Participants per group (number)***	15	15	15	
***Sex (women/men)***	10/5	12/3	10/5	0.71*
***Age (years)***	54±4	47±12	50±14	0.22^#^
***BMI (kg/m^2^)***	25.0±4.3	26.3±4.8	27.0±2.7	0.40^#^
***Fasting blood glucose (mg/mL)***	94±10	94±36	105±28	0.44^#^
***Disease duration (Years)***	N/A	8.0±7.8	6.1±9.4	0.56
***Disease Activity Score (DAS 44)***	N/A	2.08±0.93	1.84±0.74	0.43
***DMARD (yes/no)***	N/A	13/2	14/1	0.54
***Anti-CCP antibodies level (U/mL)***	N/A	93.0±192.8	42.1±41.0	0.33
***Rheumatoid Factor – IgM level (IU/mL)***	N/A	69.7±130.0	59.6±67.0	0.79
***Rheumatoid Factor – IgA level (IU/mL)***	N/A	26.6±49.4	30.9±41.5	0.80
***ESR (mm/h)***	N/A	19±17	40±76	0.31
***hsCRP (mg/L)***	N/A	4.7±4.1	3.8±2.8	0.51

DMARD: disease-modifying anti-rheumatic drug; anti-CCP: anti-cyclic citrullinated peptide antibodies; ESR: erythrocyte sedimentation rate. hsCRP: high sensitivity C-reactive protein; BMI: body mass index.

*p value calculated using Fisher exact test and ^#^p value calculated using one way ANOVA; all other p values calculated using unpaired t test.

Values are reported as mean ± SD unless specified otherwise.

### Clinical Study: Brachial Artery Reactivity

**Figure 4:**
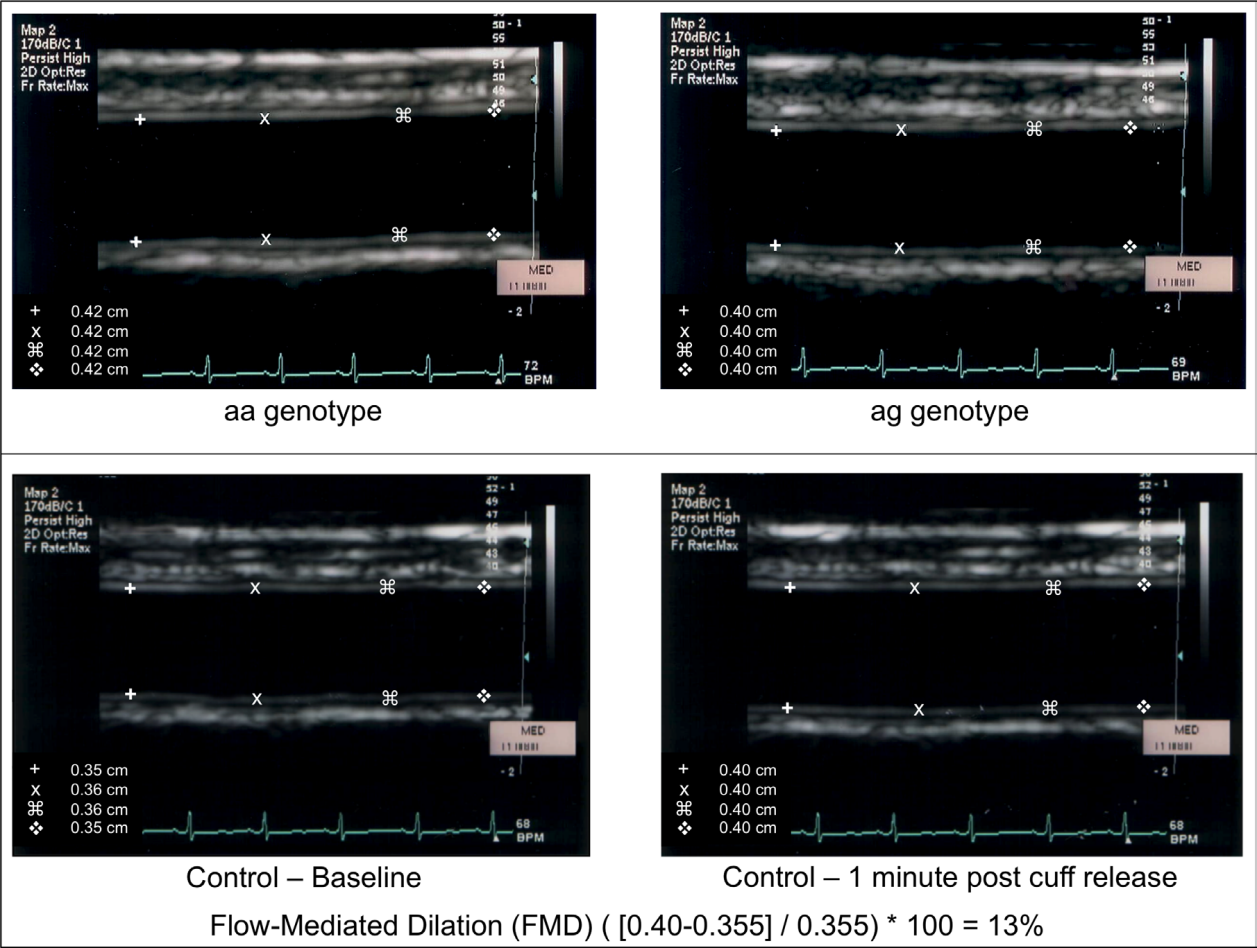
Representative images of the baseline brachial ultrasound of a participant with the aa genotype (top left picture), of a participant with the ag genotype (top right picture), and of the baseline and post-hyperemic ultrasound of a healthy control (bottom pictures). The calculation of flow-mediated dilation (FMD) using the values collected in the healthy control is also reported.

**Figure 5:**
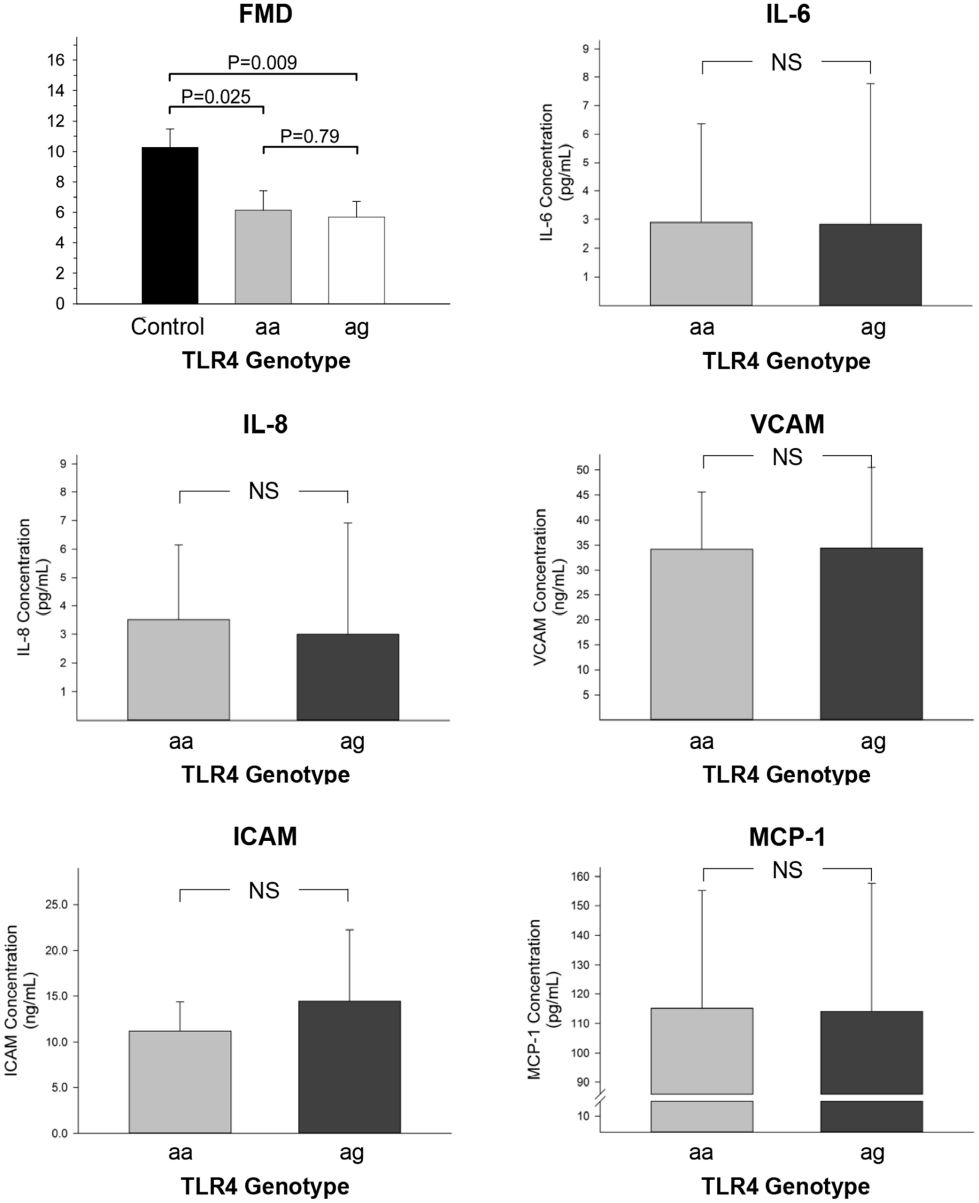
Flow-mediated dilation (top left panel) and plasma levels of IL-6 (top right panel), IL-8 (middle left panel), VCAM (middle right panel), ICAM (bottom left panel), and MCP-1 (bottom right panel) according to TLR4 genotype in study participants. Values reported as mean±SD.

Baseline brachial artery diameter was similar in the three groups (3.7±0.6 mm, 3.3±0.5 mm, and 3.4±0.7 mm in the control, aa, and ag group, respectively, p = 0.18). Flow-mediated dilation was significantly higher in the controls compared to the RA groups (p = 0.014). However, no significant differences in FMD were observed between the two patient groups (p>0.05) (figure 4 and figure 5, top left panel).

### Clinical Study: Plasma Cytokines and Adhesion Molecules in RA Patients

Plasma levels of IL-6 and IL-8, of MCP-1, and of VCAM and ICAM were similar between the aa and ag groups (figure 5).

## Discussion

The main results of our in-vitro investigations are that, in HAEC, TLR4 antagonism with eritoran: 1) inhibits LPS-induced mRNA expression of the inflammatory cytokines IL-6, IL-8, TNFα, and CCL-2, and of the adhesion molecules VCAM and ICAM; 2) inhibits ox-PAPC-induced mRNA expression of IL-8 and of IL-6, albeit to a borderline significant level; and 3) reduces LPS-induced phosphorylation of NF-κB p65 subunit and IκB-α. These findings indicate that TLR4 induces activation of human macrovascular endothelial cells through NF-κB-dependent pathways, leading to the expression of genes regulating the production of inflammatory mediators and adhesion molecules. Importantly, TLR4-dependent activation of these pathways is not restricted to exogenous ligands such as LPS, as mRNA expression of IL-6 and IL-8 was also triggered by Ox-PAPC. These phospholipid oxidation products are present in sites of chronic inflammation, in apoptotic cell membranes, and in oxidized LDL [26], suggesting a potential pathophysiologic role of Ox-PAPC-dependent LTR4 activation in atherosclerosis. Finally, as FFA-induced IL-6 expression was not affected by eritoran, our data suggest that TLR4 activation in macrovascular endothelial cells is possibly restricted to specific lipid ligands such as Ox-PAPC.

Our results are in agreement with the recent findings from Lu and colleagues. These authors reported that, in HAEC and in dermal microvascular endothelial cells, LPS induced an increase in mRNA expression and synthesis of IL-6, as well as a more robust gene expression of IL-8 and other inflammatory cytokines, ICAM, VCAM, chemokines, growth factors, and adhesion molecules [27]. Our study shows that also Ox-PAPC, an endogenous byproduct of phospholipid peroxidation, triggers TLR4-dependent expression of inflammatory mediators, confirming and further expanding previous observations [28]. In addition, we also confirm previous evidence that the synthesis of inflammatory mediators induced by TLR4 signaling is mediated by activation of NF-κB [4], which has been associated with endothelial injury [7]. Our in vitro findings complement the current understanding of the role of TLR4 signaling in the pathogenesis of endothelial dysfunction proposed by Liang and colleagues. [29] In a series of elegant ex-vivo experiments using vessels from type 2 diabetic mice with mutated TLR4 (TLR4^−/−^), these investigators demonstrated that TLR4 activation leads to the transcription of NADPH oxidase 1 and 4 and increased reactive oxidative species (ROS) generation. In turn, the higher concentration of ROS impairs eNOS coupling, thereby reducing nitric oxide (NO) production; blunts endothelium-derived hyperpolarizing factor-mediated vasodilation; and increases the synthesis of vasoconstrictor prostanoids by cyclooxygenase 1. Thus, activation of TLR4 in macrovascular endothelial cells appears to trigger an inflammatory and proatherosclerotic phenotype, with enhanced expression of cytokines and adhesion molecules, increased ROS production, and abnormal synthesis of vasoactive factors. Conversely, in our study, TRL4 blockade by eritoran significantly inhibited LPS-stimulated expression of inflammatory and adhesion molecules in macrovascular endothelial cell cultures. In the same setting, TRL4 signaling inhibition was also effective in blunting IL-8 responses to Ox-PAPC treatment. These findings are in keeping with previous observations of mitigated inflammatory responses to LPS in endothelial cells pretreated by TRL4-blocking antibodies [27]. Even more importantly, our results are consistent with the recent evidence that TLR4 antagonism may inhibit vascular inflammation and atherogenesis in diabetic ApoE^−/−^ mice, supporting the concept that TLR4 pathway is a potential therapeutic target [30].

The principal findings of our clinical study are that well-treated RA patients, despite low disease activity, have impaired brachial artery FMD and that the presence of the Asp299Gly TLR4 polymorphism is not associated with better endothelial function or lower plasma levels of inflammatory cytokines, adhesion molecules, and MCP-1. Of note, MCP-1 and its associated protein MCPIP are increased in experimental models of RA and exert detrimental effects on endothelial function by decreasing NO bioavailability [31]. Therefore, our investigation does not confirm the hypothesis that the Asp299Gly polymorphism, which has been reported to decrease TLR4 signaling activity [10], is associated with better vasodilator function compared to the wild type in this population. These results are apparently at odds with our and other groups’ laboratory data that suggest LTR4-mediated endothelial activation and dysfunction, and are not consistent with the evidence that the Asp299Gly TLR4 polymorphism is associated with a decreased risk of atherosclerosis [11]. A number of factors may account for our negative results. The impact of the Asp299Gly variant on the endothelium may be modest and minor differences in endothelial function may have been missed due to small sample size and high variability of FMD. Alternatively, it is possible that the effects of this polymorphism on endothelial function are negligible in patients with RA, in whom other inflammatory stimuli may represent the main players in the pathogenesis of endothelial damage and atherosclerosis [32]. In this regard, it must be considered that patients with RA recruited for our study were receiving optimal medical treatment and had normal DAS 44 scores and low levels of inflammatory markers. It might be postulated, therefore, that it was difficult under those conditions to detect possible differences in endothelial activation and vasodilator function related to polymorphisms of the TRL4 gene. However, our results are in line with other studies that did not find a significant association between TLR4 Asp299Gly polymorphism and risk for development and progression of atherosclerosis, including a recently published meta-analysis [33]–[34].

In conclusion, the results of our in vitro study indicate that TLR4 signaling in endothelial cells may be triggered by both LPS and endogenous ligands, such as oxidized phospholipids, leading to endothelial cell activation and a proinflammatory phenotype. Importantly, TLR4 activation by endogenous ligands is likely to occur in the presence of a proatherosclerotic milieu, where it can contribute to maintain and amplify endothelial activation and vessel wall inflammation. Further investigations are needed to better understand the potential impact of TLR4 polymorphisms on endothelial function in this and other populations at onset of the disease and while on high disease activity. Certainly the findings of our in vivo investigation do not suggest that the Asp299Gly TLR4 polymorphism is associated with improved endothelial function in patients with RA, while treated and in low disease activity.
